# Supplementary material for: Large-scale animal model study uncovers altered brain pH and lactate levels as a transdiagnostic endophenotype of neuropsychiatric disorders involving cognitive impairment
Source: eLife. 2024 Mar 26;12:RP89376. doi: 10.7554/eLife.89376 (PMC10965225; doi:10.7554/eLife.89376)
Supplement: Supplementary file 1. [file elife-89376-supp1.docx]

**Supplementary File1.** Animal models used in this study

|  | Name | Description | Related diseases, behaviors, or conditions |
| --- | --- | --- | --- |
| Exploratory cohort | | | |
| 1 | APP Tg | Mice expressing familial Alzheimer’s disease-mutant human amyloid beta precursor protein (PDGF-hAPP_swe/Ind_, line J20) (1) | AD (2,3) |
| 2 | Arid1b KO | Mice with heterozygous knockout of the AT-rich interaction domain 1b (4) | ASD (5,6) |
| 3 | Auts2 KO | Mice with heterozygous knockout of the Autism susceptibility candidate 2 (7) | ASD (8–10), ID (11), SZ (12) |
| 4 | Barp KO | Voltage gated calcium channel beta-anchoring and -regulatory protein KO mice (13) | Dysregulated voltage-gated Ca^2+^ channel activity (13) |
| 5 | Bdnf KO | Mice with heterozygous knockout of the brain derived neurotrophic factor^*^ (JAX stock #004339) | SZ, AD, PD (14) |
| 6 | BTBR | Inbred mouse strain BTBR T+ tf/J (15,16) | ASD (15,16) |
| 7 | Camk2a KO | Mice with heterozygous knockout of the calcium/calmodulin-dependent protein kinase II alpha (17–19) (JAX stock #002362) | BD (20–22), SZ (23) |
| 8 | Camkk1 KO | Mice with forebrain-specific constitutively active form of calcium/calmodulin kinase kinase 1 (24) | Impaired long-term memory (24) |
| 9 | Ccnd2 KO | Cyclin D2 KO mice (25) | Impaired adult brain neurogenesis (26,27) |
| 10 | CFA treatment | Mouse model of chronic inflammatory pain induced by complete Freund’s adjuvant (CFA) (28,29) | Inflammatory pain (28,29) |
| 11 | Chd8 KO | Mice with heterozygous knockout of the long isoform of chromodomain helicase DNA-binding protein 8 (30) | ASD (31–35) |
| 12 | Chn1 KO | Chimerin 1 (α-chimerin) KO mice (36) | ASD (36) |
| 13 | Clock mutant | Mice with N-ethyl-N-nitrosourea-induced mutation in circadian locomotor output cycles kaput(37,38) (JAX stock #002923) | BD (39,40), SZ (41) |
| 14 | Corticosterone treatment | Mice chronically treated with corticosterone (42,43) | MD (44–46) |
| 15 | Crmp2 KO | Collapsin response mediator protein 2 KO mice (47) | AD (48), SZ (49) |
| 16 | Dextran treatment | Mice treated with dextran sulfate sodium (50) | Colitis (50) |
| 17 | Disc1-L100P mutant | Mice with N-ethyl-N-nitrosourea-induced L100P amino acid exchange mutation in exon 2 of Disrupted-in-Schizophrenia 1 (51) | SZ (52–54) |
| 18 | Disc1-Q31L mutant | Mice with N-ethyl-N-nitrosourea-induced Q31L amino acid exchange mutation in exon 2 of Disrupted-in-Schizophrenia 1 (51) | SZ (52–54) |
| 19 | Dyrk1a KO | Mice with heterozygous knockout of the dual specificity tyrosine phosphorylation regulated kinase 1a (55) | ASD/ID (6,56,57) |
| 20 | ECS treatment | Mice treated with electroconvulsive stimulation (58,59) | Treatment for MD (60,61) |
| 21 | Fmr1 KO | Fragile X mental retardation protein translational regulator 1 KO mice (62) | ASD, FMR, SZ (23) |
| 22 | Gasc1 hypomorph | Gene amplified in squamous cell carcinoma 1 hypomorphic mutant mice (63,64) | ASD (65) |
| 23 | Glra4 KO | Glycine receptor alpha 4 KO mice (66,67) | ID (68) |
| 24 | Grin1 KO (postnatal) | GABArgic neuron-specific glutamate receptor, ionotropic, NMDA1 KO mice (Protein phosphatase 1, regulatory subunit 2-cre; Grin1^loxP/loxP^) (69) | SZ (70,71) |
| 25 | Grin1 KO (adult) | GABArgic neuron-specific glutamate receptor, ionotropic, NMDA1 KO mice (Protein phosphatase 1, regulatory subunit 2-cre; Grin1^loxP/loxP^) (69) | SZ (70,71) |
| 26 | Gunn rat | Gunn rats (Gunn/Slc-j/j) (72) | SZ (73) |
| 27 | Hivep2 KO | Human immunodeficiency virus type 1 enhancer binding protein 2 (Schnurri-2) KO mice (74) | ID (75,76), SZ (74) |
| 28 | Hyponatremia | Mice treated with 1-deamino-8-D-arginine vasopressin and fed with a liquid formula (77–79) | DS (80,81) |
| 29 | Il18 KO | Interleukin 18 KO mice (82,83) | DM (84,85) |
| 30 | Ketamine treatment | Mice treated with ketamine (86) | Psychosis (87) |
| 31 | Lurasidone treatment | Mice treated with lurasidone (88) | Atypical antipsychotic therapy (89,90) |
| 32 | Mdga1 KO | MAM domain containing glycosylphospatidylinositol anchor 1 KO mice (91) | SZ (92–94) |
| 33 | Mdga2 KO | Mice with heterozygous knockout of the MAM domain containing glycosylphospatidylinositol anchor 2 (95) | ASD (96,97) |
| 34 | Methamphetamine treatment | Mice treated with methamphetamine (98) | Psychosis (99) |
| 35 | Nhe5 KO | Na^+^/H^+^ exchanger 5 KO mice (100) |  |
| 36 | Nlgn3-R451C KI | Mice with R451C amino acid exchange mutation in neuroligin 3 (15,101) | ASD (102,103) |
| 37 | Nr3c1 Tg | Mice overexpressing glucocorticoid receptor under the Camk2a promoter^#^ | MD (104) |
| 38 | Nrgn KO | Neurogranin KO mice (105–107) | SZ (108,109) |
| 39 | Oxamate treatment | Mice treated with sodium oxamate, an inhibitor of lactate dehydrogenase | Inhibition of lactate dehydrogenase |
| 40 | Pacap KO | Pituitary adenylate cyclase-activating polypeptide KO mice (110) | MD (111), SZ (112) |
| 41 | 15q dup | Mice with a paternal duplication of human chromosome 15q11-13 (113) | ASD (114–117) |
| 42 | Phencyclidine treatment | Subchronic phencyclidine-treated mice (88,118) | SZ (119) |
| 43 | PCP+Lur | Phencyclidine (PCP)- and lurasidone (Lur)-treated mice (88,118) | Atypical antipsychotic therapy (89,90) |
| 44 | Polg1 Tg | Forebrain-specific catalytic subunit of mitochondrial DNA polymerase KO mice (120) | BD (121) |
| 45 | Ppp3r1 KO | Forebrain-specific protein phosphatase 3, regulatory subunit B, alpha isoform (calcineurin B, type 1) KO mice (122–124) | SZ (125) |
| 46 | Quinpirole treatment | Mice treated with quinpirole, a dopamine D2 receptor agonist (126) | OCD (127) |
| 47 | Reln Tg | Mice lacking the C-terminal region of Reelin (128) | ASD (129–131), BD (132), SZ (133) |
| 48 | Restraint stress | Mice exposed to chronic restraint stress (134) | CS |
| 49 | Sciatic nerve cuffing | The sciatic nerve cuffing mouse model of neuropathic pain (135,136) | Chronic pain |
| 50 | Scn2a KO | Mice with heterozygous knockout of the sodium voltage-gated channel alpha subunit 2 (137) | ASD (138,139), EP (140–142), ID (143,144) |
| 51 | Sert KO | Serotonin transporter KO mice (145) | ASD (146,147) |
| 52 | Shank2 KO | SH3 and multiple ankyrin repeat domain 2 KO mice (148) | ASD (97) |
| 53 | Shank3 KO | SH3 and multiple ankyrin repeat domain 3b KO mice (149) (JAX stock #017688) | ASD (150–152) |
| 54 | Snap25-S187A KI | Mice with S187A amino acid exchange mutation in synaptosomal-associated protein of 25 kDa (153,154) | ADHD (155–160), EP (161,162), SZ (163,164) |
| 55 | Social defeat stress (acute) | Mice exposed to social defeat stress (165,166) | Acute stress |
| 56 | Social defeat stress (chronic) | Mice exposed to social defeat stress (167,168) | CS |
| 57 | STZ treatment | Mice treated with streptozotocin (169) | DM (170) |
| 58 | STZ + restraint stress | Mice treated with streptozotocin and exposed to chronic restraint stress (134,169) | DM and CS comorbidity (171) |
| 59 | Stxbp1 KO | Mice with heterozygous knockout of the syntaxin-binding protein 1 (172) | ASD/ID (57,144,173), EP (174,175) |
| 60 | Syngap1 KO | Mice with heterozygous knockout of the synaptic Ras GTPase-activating protein 1 (176,177) | ID, SZ, ASD (97), EP (174) |
| 61 | Thalidomide treatment | Rats prenatally exposed to thalidomide (178,179) | ASD (180) |
| 62 | Tnxb KO | Tenascin X KO mice (181,182) | EDS (183), SZ (184–186) |
| 63 | Tsc1 KO | Astrocyte-specific tuberous sclerosis complex 1 KO mice (Glial fibrillary acidic protein-cre; Tsc1^loxP/loxP^) (187) | TSC (188) |
| 64 | Txn1-F54L Mut | Rats with a missense mutation in the thioredoxin 1 F51L generated by ENU-mutagenesis (189) | EP (189) |
| 65 | Valproic acid treatment | Mice prenatally exposed to valproic acid (190) | ASD (191) |
| Confirmatory cohort | | | |
| 66 | Act Tg | Mice with forebrain-specific overexpression of activin under the control of Camk2a promoter (192) | Anxiety-related behavior (192) |
| 67 | Actl6b KO | Actin-like 6B KO mice (193,194) | ASD (193) |
| 68 | Aldh3a2 KO | Aldehyde dehydrogenase 3 family member A2 KO mice (195,196) | Sjögren-Larsson syndrome (197) |
| 69 | B2m KO | Beta-2 microglobulin KO mice (JAX stock #002070) | Immune dysregulation (198) |
| 70 | Begain KO | Brain-enriched guanylate kinase-associated KO mice (199,200) | Neuropathic pain (199) |
| 71 | Caskin1 KO | Calcium/calmodulin-dependent serine protein kinase (CASK)-interacting protein 1 KO mice (201) | Enhanced nociception, anxiety-like behavior (201) |
| 72 | Cdkl5-K42R KI | Cyclin dependent kinase like 5 K42R knock-in mice^#^ |  |
| 73 | Cntn4 KO | Contactin-4 (BIG-2) KO mice (202) | Developmental delay (203) |
| 74 | Csgalnact1 KO | Chondroitin sulfate N-acetylgalactosaminyltransferase 1 KO mice (204,205) | Hyperactivity, increased acoustic startle response, altered social behavior (204) |
| 75 | Eif2a KO | Eukaryotic translation initiation factor 2A KO mice^#^ | Deficiency of noncanonical translation initiation factors (206) |
| 76 | Eif2d KO | Mice with heterozygous knockout of the eukaryotic translation initiation factor 2D KO mice^#^ | Deficiency of noncanonical translation initiation factors (206) |
| 77 | Fst Tg | Mice with forebrain-specific overexpression of follistatin under the control of Camk2a promoter (192) | Anxiety-related behavior (192) |
| 78 | GluA1-C811S KI | GluA1 palmitoylation-deficient (Cys811 to Ser substitution) knock-in mice (207,208) | Elevated seizure susceptibility, prolonged fear memory (207,208) |
| 79 | Grin1-Rgsc174 Mut | Mice with missense mutation in NMDA receptor subunit 1 (209) | Increased novelty-seeking behavior (209) |
| 80 | hMfn2-D210V Tg | Mice with pathogenic mutant of human Mitofusin 2 [hMFN2(D210V): Camk2a-tTA/TRE-hMFN2(D210V) Tg mice] (210,211) | Charcot–Marie–Tooth disease type 2A (212,213) |
| 81 | Il1r1 KO | Interleukin 1 receptor, type I KO mice (JAX stock #003245) |  |
| 82 | Isolation stress | Layer-type neonatal chicks (*Gallus gallus*) exposed to isolation-induced stress (214,215) | Isolation stress |
| 83 | LINC00643 KO | Long intergenic non-protein coding RNA 643 (1700086L19Rik) KO mice^#^ |  |
| 84 | Lis1 KO | Mice with heterozygous knockout of the platelet-activating factor acetylhydrolase, isoform 1b, subunit 1 (216,217) | Lissencephaly (218,219) |
| 85 | Ndufs4 KO | NADH:ubiquinone oxidoreductase core subunit S4 KO mice (220) | Leigh encephalopathy (221,222) |
| 86 | nSP treatment | Prenatal treatment of amorphous nanosilica particle (223) | Reproductive and developmental hazards of nanomaterials |
| 87 | Oprk1 KO | Opioid receptor kappa 1 KO mice (224) | Analgesia, addiction |
| 88 | Papst1 KO | Mice with heterozygous knockout of the 3′-phosphoadenosine 5′-phosphosulfate (PAPS) transporter^#^ |  |
| 89 | Picrotoxin treatment | Mice prenatally exposed to GABA_A_ receptor antagonist picrotoxin (225) | ASD (226) |
| 90 | Polb KO | Nex-Cre/DNA polymerase beta^fl/fl^ mice (227) | Genetic diseases related to DNA repair (228,229) |
| 91 | Prickle1 KO | Mice with heterozygous knockout of the prickle1 (230,231) | EP (232,233), ASD (234,235) |
| 92 | Prrt3 KO | Actb-Cre/proline rich transmembrane protein 3^fl/fl^ mice (236) | Orphan metabotropic receptor |
| 93 | RNG105 KO | Mice with heterozygous knockout of the cell cycle associated protein 1 (237) | ASD, Asperger’s syndrome (238) |
| 94 | Scn1a-A1783V KI | Mice carrying the sodium voltage-gated channel alpha subunit 1 A1783V mutation (239) | Dravet syndrome (240,241) |
| 95 | Scop KO | Suprachiasmatic nucleus circadian oscillatory protein KO mice (242) | Circadian regulation of memory formation (242) |
| 96 | Sleep disturbance | A mouse model of chronic sleep disorders caused by psychophysiological stress (243,244) | Sleep disorders (243,244) |
| 97 | Srr KO | Serine racemase KO mice (245,246) | SZ (247,248) |
| 98 | Stx1a-R151G KI | Syntaxin-1A (R151G) knock-in mice (249) | hyperactivity, anxiety- and depression-related behaviors, impaired working memory (249) |
| 99 | Tgm3 KO | Transglutaminase 3 KO mice (250) | ASD (251) |
| 100 | Tmem115 KO | Transmembrane protein 115 KO mice^#^ |  |
| 101 | Trpm2 KO | Transient receptor potential melastatin 2 KO mice (252) | BD (253,254) |
| 102 | Ts1Cje | Ts(16C-tel)1Cje mouse carrying a 7.6 Mb segmental trisomy of mouse chromosome 16 (255) | Down syndrome (256) |
| 103 | Tsc2 KO | Mice with heterozygous knockout of the tuberous sclerosis complex 2 (257–259) | TSC (188) |
| 104 | Ube3a^m-/p+^ | Ubiquitin protein ligase E3A maternal-deficient mice (260) | Angelman syndrome (261) |
| 105 | Vmat1-Thr/Thr KI | Mice with humanized substitutions of the vesicular monoamine transporter 1 (replacement of the 133Asn of mouse Vmat1 with Thr or Ile) (262) | BD (263), Anxiety (264,265) |
| 106 | Vmat1-Thr/Ile KI | Mice with humanized substitutions of the vesicular monoamine transporter 1 (replacement of the 133Asn of mouse Vmat1 with Thr or Ile) (262) | BD (263), Anxiety (264,265) |
| 107 | Vmat1-Ile/Ile KI | Mice with humanized substitutions of the vesicular monoamine transporter 1 (replacement of the 133Asn of mouse Vmat1 with Thr or Ile) (262) | BD (263), Anxiety (264,265) |
| 108 | Zeb2 KO | *De novo* zinc finger E-box binding homeobox 2 Δex7/+ mice (266) | Mowat–Wilson syndrome (267,268) |
| 109 | Zfhx2 KO | Zinc finger homeobox 2 KO mice (269) | Hyperactivity, anxiety- and depression-related behaviors (269) |

AD, Alzheimer’s disease; ADHD, attention-deficit/hyperactivity disorder; ASD, autism spectrum disorders; BD, bipolar disorder; CS, chronic stress; DM, diabetes mellitus; EDS, Ehlers-Danlos syndrome; DS, depression symptom; EP, epilepsy; FMR, Fragile X mental retardation; ID, intellectual disability, KI, knock-in; KO, knock out; MD, major depressive disorder; OCD, obsessive-compulsive disorder; PD, Parkinson’s disease; SZ, schizophrenia; Tg, transgenic; TSC, tuberous sclerosis complex. ^*^Mice with off-target deletion of conditional Bdnf allele derived from Bdnf^2lox^ mouse line were used. ^#^Unpublished mouse strain.

**References for Table S1**

1. Mucke L, Masliah E, Yu G-Q, Mallory M, Rockenstein EM, Tatsuno G, *et al.* (2000): High-level neuronal expression of Aβ1–42 in wild-type human amyloid protein precursor transgenic mice: synaptotoxicity without plaque formation. *J Neurosci* 20: 4050–4058.

2. Mullan M, Crawford F, Axelman K, Houlden H, Lilius L, Winblad B, Lannfelt L (1992): A pathogenic mutation for probable Alzheimer’s disease in the APP gene at the N-terminus of beta-amyloid. *Nat Genet* 1: 345–347.

3. Murrell J, Farlow M, Ghetti B, Benson MD (1991): A mutation in the amyloid precursor protein associated with hereditary Alzheimer’s disease. *Science* 254: 97–99.

4. Shibutani M, Horii T, Shoji H, Morita S, Kimura M, Terawaki N, *et al.* (2017): Arid1b haploinsufficiency causes abnormal brain gene expression and autism-related behaviors in mice. *Int J Mol Sci* 18: 1872.

5. D’Gama AM, Pochareddy S, Li M, Jamuar SS, Reiff RE, Lam A-TN, *et al.* (2015): Targeted DNA sequencing from autism spectrum disorder brains implicates multiple genetic mechanisms. *Neuron* 88: 910–917.

6. Fitzgerald TW, Gerety SS, Jones WD, van Kogelenberg M, King DA, McRae J, *et al.* (2015): Large-scale discovery of novel genetic causes of developmental disorders. *Nature* 519: 223–228.

7. Hori K, Nagai T, Shan W, Sakamoto A, Abe M, Yamazaki M, *et al.* (2015): Heterozygous disruption of autism susceptibility candidate 2 causes impaired emotional control and cognitive memory. *PLOS ONE* 10: e0145979.

8. Kalscheuer VM, FitzPatrick D, Tommerup N, Bugge M, Niebuhr E, Neumann LM, *et al.* (2007): Mutations in autism susceptibility candidate 2 (AUTS2) in patients with mental retardation. *Hum Genet* 121: 501–509.

9. Bakkaloglu B, O’Roak BJ, Louvi A, Gupta AR, Abelson JF, Morgan TM, *et al.* (2008): Molecular cytogenetic analysis and resequencing of contactin associated protein-like 2 in autism spectrum disorders. *Am J Hum Genet* 82: 165–173.

10. Sultana R, Yu C-E, Yu J, Munson J, Chen D, Hua W, *et al.* (2002): Identification of a novel gene on chromosome 7q11.2 interrupted by a translocation breakpoint in a pair of autistic twins. *Genomics* 80: 129–134.

11. Beunders G, Voorhoeve E, Golzio C, Pardo LM, Rosenfeld JA, Talkowski ME, *et al.* (2013): Exonic deletions in AUTS2 cause a syndromic form of intellectual disability and suggest a critical role for the C terminus. *Am J Hum Genet* 92: 210–220.

12. Zhang B, Xu Y-H, Wei S-G, Zhang H-B, Fu D-K, Feng Z-F, *et al.* (2014): Association study identifying a new susceptibility gene (AUTS2) for schizophrenia. *Int J Mol Sci* 15: 19406–19416.

13. Nakao A, Miki T, Shoji H, Nishi M, Takeshima H, Miyakawa T, Mori Y (2015): Comprehensive behavioral analysis of voltage-gated calcium channel beta-anchoring and -regulatory protein knockout mice. *Front Behav Neurosci* 9: 141.

14. Nagahara AH, Tuszynski MH (2011): Potential therapeutic uses of BDNF in neurological and psychiatric disorders. *Nat Rev Drug Discov* 10: 209–219.

15. Isshiki M, Tanaka S, Kuriu T, Tabuchi K, Takumi T, Okabe S (2014): Enhanced synapse remodelling as a common phenotype in mouse models of autism. *Nat Commun* 5: 4742.

16. McFarlane HG, Kusek GK, Yang M, Phoenix JL, Bolivar VJ, Crawley JN (2008): Autism-like behavioral phenotypes in BTBR T+tf/J mice. *Genes Brain Behav* 7: 152–163.

17. Yamasaki N, Maekawa M, Kobayashi K, Kajii Y, Maeda J, Soma M, *et al.* (2008): Alpha-CaMKII deficiency causes immature dentate gyrus, a novel candidate endophenotype of psychiatric disorders. *Mol Brain* 1: 6.

18. Hagihara H, Horikawa T, Nakamura HK, Umemori J, Shoji H, Kamitani Y, Miyakawa T (2016): Circadian gene circuitry predicts hyperactive behavior in a mood disorder mouse model. *Cell Rep* 14: 2784–2796.

19. Hagihara H, Horikawa T, Irino Y, Nakamura HK, Umemori J, Shoji H, *et al.* (2019): Peripheral blood metabolome predicts mood change-related activity in mouse model of bipolar disorder. *Mol Brain* 12: 107.

20. Le-Niculescu H, Kurian SM, Yehyawi N, Dike C, Patel SD, Edenberg HJ, *et al.* (2009): Identifying blood biomarkers for mood disorders using convergent functional genomics. *Mol Psychiatry* 14: 156–174.

21. Ament SA, Szelinger S, Glusman G, Ashworth J, Hou L, Akula N, *et al.* (2015): Rare variants in neuronal excitability genes influence risk for bipolar disorder. *Proc Natl Acad Sci* 112: 3576–3581.

22. Li H, Zhou D-S, Chang H, Wang L, Liu W, Dai S-X, *et al.* (2019): Interactome analyses implicated CAMK2A in the genetic predisposition and pharmacological mechanism of bipolar disorder. *J Psychiatr Res* 115: 165–175.

23. Purcell SM, Moran JL, Fromer M, Ruderfer D, Solovieff N, Roussos P, *et al.* (2014): A polygenic burden of rare disruptive mutations in schizophrenia. *Nature* 596: 185–90.

24. Kaitsuka T, Li S-T, Nakamura K, Takao K, Miyakawa T, Matsushita M (2011): Forebrain-specific constitutively active CaMKKα transgenic mice show deficits in hippocampus-dependent long-term memory. *Neurobiol Learn Mem* 96: 238–247.

25. Sicinski P, Donaher JL, Geng Y, Parker SB, Gardner H, Park MY, *et al.* (1996): Cyclin D2 is an FSH-responsive gene involved in gonadal cell proliferation and oncogenesis. *Nature* 384: 470–474.

26. Filipkowski RK, Kaczmarek L (2018): Severely impaired adult brain neurogenesis in cyclin D2 knock-out mice produces very limited phenotypic changes. *Prog Neuropsychopharmacol Biol Psychiatry* 80: 63–67.

27. Jaholkowski P, Kiryk A, Jedynak P, Abdallah NMB, Knapska E, Kowalczyk A, *et al.* (2009): New hippocampal neurons are not obligatory for memory formation; cyclin D2 knockout mice with no adult brain neurogenesis show learning. *Learn Mem* 16: 439–451.

28. Urban R, Scherrer G, Goulding EH, Tecott LH, Basbaum AI (2011): Behavioral indices of ongoing pain are largely unchanged in male mice with tissue or nerve injury-induced mechanical hypersensitivity. *PAIN* 152: 990–1000.

29. Wang X, Guan S, Liu A, Yue J, Hu L, Zhang K, *et al.* (2019): Anxiolytic effects of Formononetin in an inflammatory pain mouse model. *Mol Brain* 12: 36.

30. Katayama Y, Nishiyama M, Shoji H, Ohkawa Y, Kawamura A, Sato T, *et al.* (2016): CHD8 haploinsufficiency results in autistic-like phenotypes in mice. *Nature* 537: 675–679.

31. O’Roak BJ, Vives L, Fu W, Egertson JD, Stanaway IB, Phelps IG, *et al.* (2012): Multiplex targeted sequencing identifies recurrently mutated genes in autism spectrum disorders. *Science* 338: 1619–1622.

32. O’Roak BJ, Vives L, Girirajan S, Karakoc E, Krumm N, Coe BP, *et al.* (2012): Sporadic autism exomes reveal a highly interconnected protein network of de novo mutations. *Nature* 485: 246–250.

33. Talkowski ME, Rosenfeld JA, Blumenthal I, Pillalamarri V, Chiang C, Heilbut A, *et al.* (2012): Sequencing chromosomal abnormalities reveals neurodevelopmental loci that confer risk across diagnostic Bboundaries. *Cell* 149: 525–537.

34. Neale BM, Kou Y, Liu L, Ma’ayan A, Samocha KE, Sabo A, *et al.* (2012): Patterns and rates of exonic de novo mutations in autism spectrum disorders. *Nature* 485: 242–245.

35. Bernier R, Golzio C, Xiong B, Stessman HA, Coe BP, Penn O, *et al.* (2014): Disruptive CHD8 mutations define a subtype of autism early in development. *Cell* 158: 263–276.

36. Iwata R, Ohi K, Kobayashi Y, Masuda A, Iwama M, Yasuda Y, *et al.* (2014): RacGAP α2-chimaerin function in development adjusts cognitive ability in adulthood. *Cell Rep* 8: 1257–1264.

37. Vitaterna MH, King DP, Chang A-M, Kornhauser JM, Lowrey PL, McDonald JD, *et al.* (1994): Mutagenesis and mapping of a mouse gene, clock, essential for circadian behavior. *Science* 264: 719–725.

38. Roybal K, Theobold D, Graham A, DiNieri JA, Russo SJ, Krishnan V, *et al.* (2007): Mania-like behavior induced by disruption of CLOCK. *Proc Natl Acad Sci* 104: 6406–6411.

39. Shi J, Wittke‐Thompson JK, Badner JA, Hattori E, Potash JB, Willour VL, *et al.* (2008): Clock genes may influence bipolar disorder susceptibility and dysfunctional circadian rhythm. *Am J Med Genet B Neuropsychiatr Genet* 147B: 1047–1055.

40. Soria V, Martínez-Amorós È, Escaramís G, Valero J, Pérez-Egea R, García C, *et al.* (2010): Differential association of circadian genes with mood disorders: CRY1 and NPAS2 are associated with unipolar major depression and CLOCK and VIP with bipolar disorder. *Neuropsychopharmacology* 35: 1279–1289.

41. Kishi T, Kitajima T, Ikeda M, Yamanouchi Y, Kinoshita Y, Kawashima K, *et al.* (2009): Association study of clock gene (CLOCK) and schizophrenia and mood disorders in the Japanese population. *Eur Arch Psychiatry Clin Neurosci* 259: 293.

42. Murray F, Smith DW, Hutson PH (2008): Chronic low dose corticosterone exposure decreased hippocampal cell proliferation, volume and induced anxiety and depression like behaviours in mice. *Eur J Pharmacol* 583: 115–127.

43. Zhao Y, Ma R, Shen J, Su H, Xing D, Du L (2008): A mouse model of depression induced by repeated corticosterone injections. *Eur J Pharmacol* 581: 113–120.

44. Antonijevic IA, Steiger A (2003): Depression-like changes of the sleep-EEG during high dose corticosteroid treatment in patients with multiple sclerosis. *Psychoneuroendocrinology* 28: 780–795.

45. Brown ES, J. Woolston D, Frol A, Bobadilla L, Khan DA, Hanczyc M, *et al.* (2004): Hippocampal volume, spectroscopy, cognition, and mood in patients receiving corticosteroid therapy. *Biol Psychiatry* 55: 538–545.

46. Brown ES, Suppes T (1998): Mood symptoms during corticosteroid therapy: a review. *Harv Rev Psychiatry* 5: 239–246.

47. Nakamura H, Yamashita N, Kimura A, Kimura Y, Hirano H, Makihara H, *et al.* (2016): Comprehensive behavioral study and proteomic analyses of CRMP2-deficient mice. *Genes Cells* 21: 1059–1079.

48. Yoshida H, Watanabe A, Ihara Y (1998): Collapsin response mediator protein-2 is associated with neurofibrillary tangles in Alzheimer’s disease. *J Biol Chem* 273: 9761–9768.

49. Nakata K, Ujike H, Sakai A, Takaki M, Imamura T, Tanaka Y, Kuroda S (2003): The human dihydropyrimidinase-related protein 2 gene on chromosome 8p21 is associated with paranoid-type schizophrenia. *Biol Psychiatry* 53: 571–576.

50. Nyuyki KD, Cluny NL, Swain MG, Sharkey KA, Pittman QJ (2018): Altered brain excitability and increased anxiety in mice with experimental colitis: consideration of hyperalgesia and sex differences. *Front Behav Neurosci* 12: 58.

51. Shoji H, Toyama K, Takamiya Y, Wakana S, Gondo Y, Miyakawa T (2012): Comprehensive behavioral analysis of ENU-induced Disc1-Q31L and -L100P mutant mice. *BMC Res Notes* 5: 108.

52. St Clair D, Blackwood D, Muir W, Walker M, St Clair D, Muir W, *et al.* (1990): Association within a family of a balanced autosomal translocation with major mental illness. *The Lancet* 336: 13–16.

53. Millar JK, Wilson-Annan JC, Anderson S, Christie S, Taylor MS, Semple CAM, *et al.* (2000): Disruption of two novel genes by a translocation co-segregating with schizophrenia. *Hum Mol Genet* 9: 1415–1423.

54. Ekelund J, Hovatta I, Parker A, Paunio T, Varilo T, Martin R, *et al.* (2001): Chromosome 1 loci in Finnish schizophrenia families. *Hum Mol Genet* 10: 1611–1617.

55. Raveau M, Shimohata A, Amano K, Miyamoto H, Yamakawa K (2018): DYRK1A-haploinsufficiency in mice causes autistic-like features and febrile seizures. *Neurobiol Dis* 110: 180–191.

56. Krumm N, O’Roak BJ, Shendure J, Eichler EE (2014): A de novo convergence of autism genetics and molecular neuroscience. *Trends Neurosci* 37: 95–105.

57. McRae JF, Clayton S, Fitzgerald TW, Kaplanis J, Prigmore E, Rajan D, *et al.* (2017): Prevalence and architecture of de novo mutations in developmental disorders. *Nature* 542: 433–438.

58. Imoto Y, Segi-Nishida E, Suzuki H, Kobayashi K (2017): Rapid and stable changes in maturation-related phenotypes of the adult hippocampal neurons by electroconvulsive treatment. *Mol Brain* 10: 8.

59. Kobayashi K, Imoto Y, Yamamoto F, Kawasaki M, Ueno M, Segi-Nishida E, Suzuki H (2016): Rapid and lasting enhancement of dopaminergic modulation at the hippocampal mossy fiber synapse by electroconvulsive treatment. *J Neurophysiol* 117: 284–289.

60. Husain MM, Rush AJ, Fink M, Knapp R, Petrides G, Rummans T, *et al.* (2004): Speed of response and remission in major depressive disorder with acute Electroconvulsive therapy (ECT): A consortium for research in ECT (CORE) report. *J Clin Psychiatry* 65: 485–491.

61. Pagnin D, de Queiroz V, Pini S, Cassano GB (2004): Efficacy of ECT in depression: a meta-analytic review. *J ECT* 20: 13–20.

62. Consorthium TD-BFX, Bakker CE, Verheij C, Willemsen R, Helm R van der, Oerlemans F, *et al.* (1994): Fmr1 knockout mice: a model to study fragile X mental retardation. *Cell* 78: 23–33.

63. Taga T, Yamaguchi Y, Kokubo Y, Hattori S, Takao K, Inazawa J, *et al.* (2011): Establishment of a new mouse model of neurodevelopmental disorder. *J Pharmacol Sci* 115: 35–35.

64. Sudo G, Kagawa T, Kokubu Y, Inazawa J, Taga T (2016): Increase in GFAP-positive astrocytes in histone demethylase GASC1/KDM4C/JMJD2C hypomorphic mutant mice. *Genes Cells* 21: 218–225.

65. Kantojärvi K, Onkamo P, Vanhala R, Alen R, Hedman M, Sajantila A, *et al.* (2010): Analysis of 9p24 and 11p12-13 regions in autism spectrum disorders: rs1340513 in the JMJD2C gene is associated with ASDs in Finnish sample. *Psychiatr Genet* 20: 102–108.

66. Nishizono H, Darwish M, Endo TA, Uno K, Abe H, Yasuda R (2020): Glycine receptor α4 subunit facilitates the early embryonic development in mice. *Reproduction* 159: 41.

67. Darwish M, Endo T, Uno K, Takao K, Nishizono H (2019): Investigation into the role of Glra4, a functionally unknown subunit of glycine receptors, in brain function and neurological disorders. *42nd Annu Meet Jpn Neurosci Soc* Abstract.

68. Labonne JDJ, Graves TD, Shen Y, Jones JR, Kong I-K, Layman LC, Kim H-G (2016): A microdeletion at Xq22.2 implicates a glycine receptor GLRA4 involved in intellectual disability, behavioral problems and craniofacial anomalies. *BMC Neurol* 16: 132.

69. Belforte JE, Zsiros V, Sklar ER, Jiang Z, Yu G, Li Y, *et al.* (2010): Postnatal NMDA receptor ablation in corticolimbic interneurons confers schizophrenia-like phenotypes. *Nat Neurosci* 13: 76–83.

70. Begni S, Moraschi S, Bignotti S, Fumagalli F, Rillosi L, Perez J, Gennarelli M (2003): Association between the G1001C polymorphism in the GRIN1 gene promoter region and schizophrenia. *Biol Psychiatry* 53: 617–619.

71. Zhao X, Li H, Shi Y, Tang R, Chen W, Liu J, *et al.* (2006): Significant association between the genetic variations in the 5′ end of the N-Methyl-D-Aspartate receptor subunit gene GRIN1 and schizophrenia. *Biol Psychiatry* 59: 747–753.

72. Hayashida M, Miyaoka T, Tsuchie K, Yasuda H, Wake R, Nishida A, *et al.* (2009): Hyperbilirubinemia-related behavioral and neuropathological changes in rats: A possible schizophrenia animal model. *Prog Neuropsychopharmacol Biol Psychiatry* 33: 581–588.

73. Müller N, Schiller P, Ackenheil M (1991): Coincidence of schizophrenia and hyperbilirubinemia. *Pharmacopsychiatry* 24: 225–228.

74. Takao K, Kobayashi K, Hagihara H, Ohira K, Shoji H, Hattori S, *et al.* (2013): Deficiency of Schnurri-2, an MHC enhancer binding protein, induces mild chronic inflammation in the brain and confers molecular, neuronal, and behavioral phenotypes related to schizophrenia. *Neuropsychopharmacology* 38: 1409–1425.

75. Srivastava S, Engels H, Schanze I, Cremer K, Wieland T, Menzel M, *et al.* (2016): Loss-of-function variants in *HIVEP2* are a cause of intellectual disability. *Eur J Hum Genet* 24: 556.

76. Steinfeld H, Cho MT, Retterer K, Person R, Schaefer GB, Danylchuk N, *et al.* (2016): Mutations in HIVEP2 are associated with developmental delay, intellectual disability, and dysmorphic features. *Neurogenetics* 17: 159–164.

77. Fujisawa H, Sugimura Y, Takagi H, Mizoguchi H, Takeuchi H, Izumida H, *et al.* (2015): Chronic hyponatremia causes neurologic and psychologic impairments. *J Am Soc Nephrol* ASN.2014121196.

78. Izumida H, Takagi H, Fujisawa H, Iwata N, Nakashima K, Takeuchi S, *et al.* (2017): NMDA receptor antagonist prevents cell death in the hippocampal dentate gyrus induced by hyponatremia accompanying adrenal insufficiency in rats. *Exp Neurol* 287, Part 1: 65–74.

79. Kawakami T, Fujisawa H, Nakayama S, Yoshino Y, Hattori S, Seino Y, *et al.* (2020): Vasopressin escape and memory impairment in a model of chronic syndrome of inappropriate secretion of antidiuretic hormone in mice. *Endocr J* 68: 31–43.

80. Fan S-S, Lin L-F, Chen VC-H, Hsieh C-W, Hsiao H-P, McIntyre RS, *et al.* (2020): Effects of lower past-year serum sodium and hyponatremia on depression symptoms and cognitive impairments in patients with hemodialysis. *Ther Apher Dial* 24: 169–177.

81. Fujisawa C, Umegaki H, Sugimoto T, Samizo S, Huang CH, Fujisawa H, *et al.* (2021): Mild hyponatremia is associated with low skeletal muscle mass, physical function impairment, and depressive mood in the elderly. *BMC Geriatr* 21: 15.

82. Yamanishi K, Doe N, Mukai K, Ikubo K, Hashimoto T, Uwa N, *et al.* (2019): Interleukin-18-deficient mice develop hippocampal abnormalities related to possible depressive-like behaviors. *Neuroscience* 408: 147–160.

83. Yamanishi K, Hashimoto T, Miyauchi M, Mukai K, Ikubo K, Uwa N, *et al.* (2020): Analysis of genes linked to depressive‑like behaviors in interleukin‑18‑deficient mice: Gene expression profiles in the brain. *Biomed Rep* 12: 3–10.

84. Kretowski A, Mironczuk K, Karpinska A, Bojaryn U, Kinalski M, Puchalski Z, Kinalska I (2002): Interleukin-18 promoter polymorphisms in type 1 diabetes. *Diabetes* 51: 3347–3349.

85. Netea MG, Joosten LAB, Lewis E, Jensen DR, Voshol PJ, Kullberg BJ, *et al.* (2006): Deficiency of interleukin-18 in mice leads to hyperphagia, obesity and insulin resistance [no. 6]. *Nat Med* 12: 650–656.

86. Chatterjee M, Ganguly S, Srivastava M, Palit G (2011): Effect of ‘chronic’ versus ‘acute’ ketamine administration and its ‘withdrawal’ effect on behavioural alterations in mice: Implications for experimental psychosis. *Behav Brain Res* 216: 247–254.

87. Lahti AC, Weiler MA, Tamara Michaelidis BA, Parwani A, Tamminga CA (2001): Effects of ketamine in normal and schizophrenic volunteers. *Neuropsychopharmacology* 25: 455–467.

88. Huang M, Kwon S, Rajagopal L, He W, Meltzer HY (2018): 5-HT1A parital agonism and 5-HT7 antagonism restore episodic memory in subchronic phencyclidine-treated mice: role of brain glutamate, dopamine, acetylcholine and GABA. *Psychopharmacology (Berl)* 235: 2795–2808.

89. Meltzer HY, Cucchiaro J, Silva R, Ogasa M, Phillips D, Xu J, *et al.* (2011): Lurasidone in the treatment of schizophrenia: a randomized, double-blind, placebo- and olanzapine-controlled study. *Am J Psychiatry* 168: 957–967.

90. Ishibashi T, Horisawa T, Tokuda K, Ishiyama T, Ogasa M, Tagashira R, *et al.* (2010): Pharmacological profile of lurasidone, a novel antipsychotic agent with potent 5-hydroxytryptamine 7 (5-HT7) and 5-HT1A receptor activity. *J Pharmacol Exp Ther* 334: 171–181.

91. Connor SA, Ammendrup-Johnsen I, Kishimoto Y, Karimi Tari P, Cvetkovska V, Harada T, *et al.* (2017): Loss of synapse repressor MDGA1 enhances perisomatic inhibition, confers resistance to network excitation, and impairs cognitive function. *Cell Rep* 21: 3637–3645.

92. Kähler AK, Djurovic S, Kulle B, Jönsson EG, Agartz I, Hall H, *et al.* (2008): Association analysis of schizophrenia on 18 genes involved in neuronal migration: MDGA1 as a new susceptibility gene. *Am J Med Genet B Neuropsychiatr Genet* 147B: 1089–1100.

93. Li J, Liu J, Feng G, Li T, Zhao Q, Li Y, *et al.* (2011): The MDGA1 gene confers risk to schizophrenia and bipolar disorder. *Schizophr Res* 125: 194–200.

94. Hossain MR, Jamal M, Tanoue Y, Ojima D, Takahashi H, Kubota T, *et al.* (2020): MDGA1-deficiency attenuates prepulse inhibition with alterations of dopamine and serotonin metabolism: An ex vivo HPLC-ECD analysis. *Neurosci Lett* 716: 134677.

95. Connor SA, Ammendrup-Johnsen I, Chan AW, Kishimoto Y, Murayama C, Kurihara N, *et al.* (2016): Altered cortical dynamics and cognitive function upon haploinsufficiency of the autism-linked excitatory synaptic suppressor MDGA2. *Neuron* 91: 1052–1068.

96. Bucan M, Abrahams BS, Wang K, Glessner JT, Herman EI, Sonnenblick LI, *et al.* (2009): Genome-wide analyses of exonic copy number variants in a family-based study point to novel autism susceptibility genes. *PLOS Genet* 5: e1000536.

97. Pinto D, Pagnamenta AT, Klei L, Anney R, Merico D, Regan R, *et al.* (2010): Functional impact of global rare copy number variation in autism spectrum disorders. *Nature* 466: 368–372.

98. McClay JL, Adkins DE, Vunck SA, Batman AM, Vann RE, Clark SL, *et al.* (2013): Large-scale neurochemical metabolomics analysis identifies multiple compounds associated with methamphetamine exposure. *Metabolomics* 9: 392–402.

99. Glasner-Edwards S, Mooney LJ (2014): Methamphetamine psychosis: epidemiology and management. *CNS Drugs* 28: 1115–1126.

100. Togashi K, Wakatsuki S, Furuno A, Tokunaga S, Nagai Y, Araki T (2013): Na+/H+ exchangers induce autophagy in neurons and inhibit polyglutamine-induced aggregate formation. *PLOS ONE* 8: e81313.

101. Tabuchi K, Blundell J, Etherton MR, Hammer RE, Liu X, Powell CM, Südhof TC (2007): A neuroligin-3 mutation implicated in autism increases inhibitory synaptic transmission in mice. *Science* 318: 71–76.

102. Jamain S, Quach H, Betancur C, Råstam M, Colineaux C, Gillberg IC, *et al.* (2003): Mutations of the X-linked genes encoding neuroligins NLGN3 and NLGN4 are associated with autism. *Nat Genet* 34: 27–29.

103. Südhof TC (2008): Neuroligins and neurexins link synaptic function to cognitive disease. *Nature* 455: 903–911.

104. van West D, Van Den Eede F, Del-Favero J, Souery D, Norrback K-F, Van Duijn C, *et al.* (2006): Glucocorticoid receptor gene-based SNP analysis in patients with recurrent major depression. *Neuropsychopharmacology* 31: 620–627.

105. Huang FL, Huang K-P (2012): Methylphenidate improves the behavioral and cognitive deficits of neurogranin knockout mice. *Genes Brain Behav* 11: 794–805.

106. Huang FL, Huang K-P, Wu J, Boucheron C (2006): Environmental enrichment enhances neurogranin expression and hippocampal learning and memory but fails to rescue the impairments of neurogranin null mutant mice. *J Neurosci* 26: 6230–6237.

107. Pak JH, Huang FL, Li J, Balschun D, Reymann KG, Chiang C, *et al.* (2000): Involvement of neurogranin in the modulation of calcium/calmodulin-dependent protein kinase II, synaptic plasticity, and spatial learning: A study with knockout mice. *Proc Natl Acad Sci* 97: 11232–11237.

108. Stefansson H, Ophoff RA, Steinberg S, Andreassen OA, Cichon S, Rujescu D, *et al.* (2009): Common variants conferring risk of schizophrenia. *Nature* 460: 744–747.

109. Schizophrenia Working Group of the Psychiatric Genomics Consortium (2014): Biological insights from 108 schizophrenia-associated genetic loci. *Nature* 511: 421–427.

110. Hashimoto H, Shintani N, Tanaka K, Mori W, Hirose M, Matsuda T, *et al.* (2001): Altered psychomotor behaviors in mice lacking pituitary adenylate cyclase-activating polypeptide (PACAP). *Proc Natl Acad Sci* 98: 13355–13360.

111. Hashimoto R, Hashimoto H, Shintani N, Ohi K, Hori H, Saitoh O, *et al.* (2010): Possible association between the pituitary adenylate cyclase-activating polypeptide (PACAP) gene and major depressive disorder. *Neurosci Lett* 468: 300–302.

112. Hashimoto R, Hashimoto H, Shintani N, Chiba S, Hattori S, Okada T, *et al.* (2007): Pituitary adenylate cyclase-activating polypeptide is associated with schizophrenia. *Mol Psychiatry* 12: 1026–1032.

113. Nakatani J, Tamada K, Hatanaka F, Ise S, Ohta H, Inoue K, *et al.* (2009): Abnormal behavior in a chromosome- engineered mouse model for human 15q11-13 duplication seen in autism. *Cell* 137: 1235–1246.

114. Bolton PF, Veltman MWM, Weisblatt E, Holmes JR, Thomas NS, Youings SA, *et al.* (2004): Chromosome 15q11-13 abnormalities and other medical conditions in individuals with autism spectrum disorders. *Psychiatr Genet* 14: 131–137.

115. Cook Jr EH, Scherer SW (2008): Copy-number variations associated with neuropsychiatric conditions. *Nature* 455: 919–923.

116. Dykens EM, Sutcliffe JS, Levitt P (2004): Autism and 15q11-q13 disorders: Behavioral, genetic, and pathophysiological issues. *Ment Retard Dev Disabil Res Rev* 10: 284–291.

117. Takumi T, Tamada K (2018): CNV biology in neurodevelopmental disorders. *Curr Opin Neurobiol* 48: 183–192.

118. Meltzer HY, Rajagopal L, Huang M, Oyamada Y, Kwon S, Horiguchi M (2013): Translating the N-methyl-d-aspartate receptor antagonist model of schizophrenia to treatments for cognitive impairment in schizophrenia. *Int J Neuropsychopharmacol* 16: 2181–2194.

119. Peterson RC, Stillman RC (1978): Phencyclidine: An overview. Phencyclidine Abuse: An Appraisal (ed. by Petersen, R.C., Stillman, R.C.). *NIDA Res Monogr 21 US Gov Print Off Wash DC* 1–17.

120. Kasahara T, Takata A, Kato TM, Kubota-Sakashita M, Sawada T, Kakita A, *et al.* (2016): Depression-like episodes in mice harboring mtDNA deletions in paraventricular thalamus. *Mol Psychiatry* 21: 39–48.

121. Kasahara T, Ishiwata M, Kakiuchi C, Fuke S, Iwata N, Ozaki N, *et al.* (2017): Enrichment of deleterious variants of mitochondrial DNA polymerase gene (POLG1) in bipolar disorder. *Psychiatry Clin Neurosci* 71: 518–529.

122. Zeng H, Chattarji S, Barbarosie M, Rondi-Reig L, Philpot BD, Miyakawa T, *et al.* (2001): Forebrain-specific calcineurin knockout selectively impairs bidirectional synaptic plasticity and working/episodic-like memory. *Cell* 107: 617–629.

123. Miyakawa T, Leiter LM, Gerber DJ, Gainetdinov RR, Sotnikova TD, Zeng H, *et al.* (2003): Conditional calcineurin knockout mice exhibit multiple abnormal behaviors related to schizophrenia. *Proc Natl Acad Sci* 100: 8987–8992.

124. Hagihara H, Shoji H, Kuroiwa M, Graef IA, Crabtree GR, Nishi A, Miyakawa T (2022): Forebrain-specific conditional calcineurin deficiency induces dentate gyrus immaturity and hyper-dopaminergic signaling in mice. *Mol Brain* 15: 94.

125. Gerber DJ, Hall D, Miyakawa T, Demars S, Gogos JA, Karayiorgou M, Tonegawa S (2003): Evidence for association of schizophrenia with genetic variation in the 8p21.3 gene, PPP3CC, encoding the calcineurin gamma subunit. *Proc Natl Acad Sci* 100: 8993–8998.

126. Asaoka N, Nishitani N, Kinoshita H, Nagai Y, Hatakama H, Nagayasu K, *et al.* (2019): An adenosine A2A receptor antagonist improves multiple symptoms of repeated quinpirole-induced psychosis. *eNeuro* 6. https://doi.org/10.1523/ENEURO.0366-18.2019

127. Stuchlik A, Radostová D, Hatalova H, Vales K, Nekovarova T, Koprivova J, *et al.* (2016): Validity of quinpirole sensitization rat model of OCD: linking evidence from animal and clinical studies. *Front Behav Neurosci* 10: 209.

128. Sakai K, Shoji H, Kohno T, Miyakawa T, Hattori M (2016): Mice that lack the C-terminal region of Reelin exhibit behavioral abnormalities related to neuropsychiatric disorders. *Sci Rep* 6: 28636.

129. Persico AM, D’Agruma L, Maiorano N, Totaro A, Militerni R, Bravaccio C, *et al.* (2001): Reelin gene alleles and haplotypes as a factor predisposing to autistic disorder. *Mol Psychiatry* 6: 150–159.

130. Serajee FJ, Zhong H, Mahbubul Huq AHM (2006): Association of Reelin gene polymorphisms with autism. *Genomics* 87: 75–83.

131. Zhang H, Liu X, Zhang C, Mundo E, Macciardi F, Grayson DR, *et al.* (2002): Reelin gene alleles and susceptibility to autism spectrum disorders. *Mol Psychiatry* 7: 1012–1017.

132. Goes FS, Willour VL, Zandi PP, Belmonte PL, MacKinnon DF, Mondimore FM, *et al.* (2010): Sex-specific association of the reelin gene with bipolar disorder. *Am J Med Genet B Neuropsychiatr Genet* 153B: 549–553.

133. Shifman S, Johannesson M, Bronstein M, Chen SX, Collier DA, Craddock NJ, *et al.* (2008): Genome-wide association identifies a common variant in the Reelin gene that increases the risk of schizophrenia only in women. *PLOS Genet* 4: e28.

134. Shoji H, Miyakawa T (2020): Differential effects of stress exposure via two types of restraint apparatuses on behavior and plasma corticosterone level in inbred male BALB/cAJcl mice. *Neuropsychopharmacol Rep* 40: 73–84.

135. Sellmeijer J, Mathis V, Hugel S, Li X-H, Song Q, Chen Q-Y, *et al.* (2018): Hyperactivity of anterior cingulate cortex areas 24a/24b drives chronic pain-induced anxiodepressive-like consequences. *J Neurosci* 38: 3102–3115.

136. Yalcin I, Megat S, Barthas F, Waltisperger E, Kremer M, Salvat E, Barrot M (2014): The sciatic nerve cuffing model of neuropathic pain in mice. *J Vis Exp JoVE* 51608.

137. Tatsukawa T, Raveau M, Ogiwara I, Hattori S, Miyamoto H, Mazaki E, *et al.* (2019): Scn2a haploinsufficient mice display a spectrum of phenotypes affecting anxiety, sociability, memory flexibility and ampakine CX516 rescues their hyperactivity. *Mol Autism* 10: 15.

138. Buxbaum JD, Daly MJ, Devlin B, Lehner T, Roeder K, State MW (2012): The autism sequencing consortium: large-scale, high-throughput sequencing in autism spectrum disorders. *Neuron* 76: 1052–1056.

139. Tavassoli T, Kolevzon A, Wang AT, Curchack-Lichtin J, Halpern D, Schwartz L, *et al.* (2014): De novo SCN2A splice site mutation in a boy with Autism spectrum disorder. *BMC Med Genet* 15: 35.

140. Allen AS, Berkovic SF, Cossette P, Delanty N, Dlugos D, Eichler EE, *et al.* (2013): De novo mutations in epileptic encephalopathies. *Nature* 501: 217–221.

141. Heron SE, Crossland KM, Andermann E, Phillips HA, Hall AJ, Bleasel A, *et al.* (2002): Sodium-channel defects in benign familial neonatal-infantile seizures. *The Lancet* 360: 851–852.

142. Sugawara T, Tsurubuchi Y, Agarwala KL, Ito M, Fukuma G, Mazaki-Miyazaki E, *et al.* (2001): A missense mutation of the Na+ channel αII subunit gene Nav1.2 in a patient with febrile and afebrile seizures causes channel dysfunction. *Proc Natl Acad Sci* 98: 6384–6389.

143. de Ligt J, Willemsen MH, van Bon BWM, Kleefstra T, Yntema HG, Kroes T, *et al.* (2012): Diagnostic exome sequencing in persons with severe intellectual disability. *N Engl J Med* 367: 1921–1929.

144. Rauch A, Wieczorek D, Graf E, Wieland T, Endele S, Schwarzmayr T, *et al.* (2012): Range of genetic mutations associated with severe non-syndromic sporadic intellectual disability: an exome sequencing study. *The Lancet* 380: 1674–1682.

145. Tanaka M, Sato A, Kasai S, Hagino Y, Kotajima-Murakami H, Kashii H, *et al.* (2018): Brain hyperserotonemia causes autism-relevant social deficits in mice. *Mol Autism* 9: 60.

146. Bacchelli E, Maestrini E (2006): Autism spectrum disorders: Molecular genetic advances. *Am J Med Genet C Semin Med Genet* 142C: 13–23.

147. Wiggins JL, Swartz JR, Martin DM, Lord C, Monk CS (2014): Serotonin transporter genotype impacts amygdala habituation in youth with autism spectrum disorders. *Soc Cogn Affect Neurosci* 9: 832–838.

148. Won H, Lee H-R, Gee HY, Mah W, Kim J-I, Lee J, *et al.* (2012): Autistic-like social behaviour in *Shank2*-mutant mice improved by restoring NMDA receptor function. *Nature* 486: 261–265.

149. Peça J, Feliciano C, Ting JT, Wang W, Wells MF, Venkatraman TN, *et al.* (2011): Shank3 mutant mice display autistic-like behaviours and striatal dysfunction. *Nature* 472: 437–442.

150. Durand CM, Betancur C, Boeckers TM, Bockmann J, Chaste P, Fauchereau F, *et al.* (2007): Mutations in the gene encoding the synaptic scaffolding protein SHANK3 are associated with autism spectrum disorders. *Nat Genet* 39: 25–27.

151. Gauthier J, Spiegelman D, Piton A, Lafrenière RG, Laurent S, St‐Onge J, *et al.* (2009): Novel de novo SHANK3 mutation in autistic patients. *Am J Med Genet B Neuropsychiatr Genet* 150B: 421–424.

152. Moessner R, Marshall CR, Sutcliffe JS, Skaug J, Pinto D, Vincent J, *et al.* (2007): Contribution of SHANK3 mutations to autism spectrum disorder. *Am J Hum Genet* 81: 1289–1297.

153. Kataoka M, Yamamori S, Suzuki E, Watanabe S, Sato T, Miyaoka H, *et al.* (2011): A single amino acid mutation in SNAP-25 induces anxiety-related behavior in mouse. *PLOS ONE* 6: e25158.

154. Ohira K, Kobayashi K, Toyama K, Nakamura HK, Shoji H, Takao K, *et al.* (2013): Synaptosomal-associated protein 25 mutation induces immaturity of the dentate granule cells of adult mice. *Mol Brain* 6: 12.

155. Barr CL, Feng Y, Wigg K, Bloom S, Roberts W, Malone M, *et al.* (2000): Identification of DNA variants in the SNAP-25 gene and linkage study of these polymorphisms and attention-deficit hyperactivity disorder. *Mol Psychiatry* 5: 405–409.

156. Brophy K, Hawi Z, Kirley A, Fitzgerald M, Gill M (2002): Synaptosomal-associated protein 25 (SNAP-25) and attention deficit hyperactivity disorder (ADHD): evidence of linkage and association in the Irish population. *Mol Psychiatry* 7: 913–917.

157. Mill J, Curran S, Kent L, Gould A, Huckett L, Richards S, *et al.* (2002): Association study of a SNAP-25 microsatellite and attention deficit hyperactivity disorder. *Am J Med Genet* 114: 269–271.

158. Kustanovich V, Merriman B, McGough J, McCracken JT, Smalley SL, Nelson SF (2003): Biased paternal transmission of SNAP-25 risk alleles in attention-deficit hyperactivity disorder. *Mol Psychiatry* 8: 309–315.

159. Mill J, Richards S, Knight J, Curran S, Taylor E, Asherson P (2004): Haplotype analysis of SNAP-25 suggests a role in the aetiology of ADHD. *Mol Psychiatry* 9: 801–810.

160. Feng Y, Crosbie J, Wigg K, Pathare T, Ickowicz A, Schachar R, *et al.* (2005): The SNAP25 gene as a susceptibility gene contributing to attention-deficit hyperactivity disorder. *Mol Psychiatry* 10: 998–1005.

161. Hamdan FF, Myers CT, Cossette P, Lemay P, Spiegelman D, Laporte AD, *et al.* (2017): High rate of recurrent de novo mutations in developmental and epileptic encephalopathies. *Am J Hum Genet* 101: 664–685.

162. Heyne HO, Singh T, Stamberger H, Abou Jamra R, Caglayan H, Craiu D, *et al.* (2018): De novo variants in neurodevelopmental disorders with epilepsy. *Nat Genet* 50: 1048–1053.

163. Ayalew M, Le-Niculescu H, Levey DF, Jain N, Changala B, Patel SD, *et al.* (2012): Convergent functional genomics of schizophrenia: from comprehensive understanding to genetic risk prediction. *Mol Psychiatry* 17: 887–905.

164. Houenou J, Boisgontier J, Henrion A, d’Albis M-A, Dumaine A, Linke J, *et al.* (2017): A multilevel functional study of a SNAP25 at-risk variant for bipolar disorder and schizophrenia. *J Neurosci* 37: 10389–10397.

165. Kollack‐Walker, Don, Watson, Akil (1999): Differential expression of c‐fos mRNA within neurocircuits of male hamsters exposed to acute or chronic defeat. *J Neuroendocrinol* 11: 547–559.

166. Martinez M, Phillips PJ, Herbert J (1998): Adaptation in patterns of c-fos expression in the brain associated with exposure to either single or repeated social stress in male rats. *Eur J Neurosci* 10: 20–33.

167. Golden SA, Covington HE, Berton O, Russo SJ (2011): A standardized protocol for repeated social defeat stress in mice. *Nat Protoc* 6: 1183–1191.

168. Toyoda A (2017): Social defeat models in animal science: What we have learned from rodent models. *Anim Sci J* 88: 944–952.

169. Furman BL (2015): Streptozotocin-induced diabetic models in mice and rats. *Curr Protoc Pharmacol* 70: 5.47.1-5.47.20.

170. Lenzen S (2008): The mechanisms of alloxan- and streptozotocin-induced diabetes. *Diabetologia* 51: 216–226.

171. Anderson RJ, Freedland KE, Clouse RE, Lustman PJ (2001): The prevalence of comorbid depression in adults with diabetes: a meta-analysis. *Diabetes Care* 24: 1069–1078.

172. Miyamoto H, Shimohata A, Abe M, Abe T, Mazaki E, Amano K, *et al.* (2017): Potentiation of excitatory synaptic transmission ameliorates aggression in mice with Stxbp1 haploinsufficiency. *Hum Mol Genet* 26: 4961–4974.

173. Hoischen A, Krumm N, Eichler EE (2014): Prioritization of neurodevelopmental disease genes by discovery of new mutations. *Nat Neurosci* 17: 764–772.

174. Carvill GL, Heavin SB, Yendle SC, McMahon JM, O’Roak BJ, Cook J, *et al.* (2013): Targeted resequencing in epileptic encephalopathies identifies de novo mutations in CHD2 and SYNGAP1. *Nat Genet* 45: 825–830.

175. Saitsu H, Kato M, Mizuguchi T, Hamada K, Osaka H, Tohyama J, *et al.* (2008): De novo mutations in the gene encoding STXBP1 (MUNC18-1) cause early infantile epileptic encephalopathy. *Nat Genet* 40: 782–788.

176. Komiyama NH, Watabe AM, Carlisle HJ, Porter K, Charlesworth P, Monti J, *et al.* (2002): SynGAP regulates ERK/MAPK signaling, synaptic plasticity, and learning in the complex with postsynaptic density 95 and NMDA receptor. *J Neurosci* 22: 9721–9732.

177. Nakajima R, Takao K, Hattori S, Shoji H, Komiyama NH, Grant SGN, Miyakawa T (2019): Comprehensive behavioral analysis of heterozygous Syngap1 knockout mice. *Neuropsychopharmacol Rep* 39: 223–237.

178. Narita N, Kato M, Tazoe M, Miyazaki K, Narita M, Okado N (2002): Increased monoamine concentration in the brain and blood of fetal thalidomide- and valproic acid–exposed rat: Putative animal models for autism. *Pediatr Res* 52: 576–579.

179. Tsugiyama LE, Ida‐Eto M, Ohkawara T, Noro Y, Narita M (2020): Altered neuronal activity in the auditory brainstem following sound stimulation in thalidomide-induced autism model rats. *Congenit Anom* 60: 82–86.

180. Strömland K, Nordin V, Miller M, Akerström B, Gillberg C (1994): Autism in thalidomide embryopathy: a population study. *Dev Med Child Neurol* 36: 351–356.

181. Okuda-Ashitaka E, Kakuchi Y, Kakumoto H, Yamanishi S, Kamada H, Yoshidu T, *et al.* (2020): Mechanical allodynia in mice with tenascin-X deficiency associated with Ehlers-Danlos syndrome. *Sci Rep* 10: 6569.

182. Kawakami K, Matsumoto K (2011): Behavioral alterations in mice lacking the gene for tenascin-X. *Biol Pharm Bull* 34: 590–593.

183. Burch GH, Gong Y, Liu W, Dettman RW, Curry CJ, Smith L, *et al.* (1997): Tenascin–X deficiency is associated with Ehlers–Danlos syndrome. *Nat Genet* 17: 104–108.

184. Tochigi M, Zhang X, Ohashi J, Hibino H, Otowa T, Rogers M, *et al.* (2007): Association study between the TNXB locus and schizophrenia in a Japanese population. *Am J Med Genet B Neuropsychiatr Genet* 144B: 305–309.

185. Wang J, Sun S, Zhang L, Wang Z, Ye L, Liu L, *et al.* (2011): Further study of genetic association between the TNXB locus and schizophrenia. *Psychiatr Genet* 21: 216.

186. Wei J, Hemmings GP (2004): TNXB locus may be a candidate gene predisposing to schizophrenia. *Am J Med Genet B Neuropsychiatr Genet* 125B: 43–49.

187. Shimada T, Sugiura H, Yamagata K (n.d.): Inhibition of Rheb improved abnormal social behavior in astrocyte-specific Tsc1 knockout mice. *NEURO2019 42nd Annu Meet Jpn Neurosci Soc 62nd Annu Meet Jpn Soc Neurochem* PB-126.

188. Crino PB, Nathanson KL, Henske EP (2006): The tuberous sclerosis complex. *N Engl J Med* 355: 1345–1356.

189. Ohmori I, Ouchida M, Shinohara M, Kobayashi K, Ishida S, Mashimo T (2022): Novel animal model of combined generalized and focal epilepsy. *Epilepsia* 63: e80–e85.

190. Kotajima-Murakami H, Kobayashi T, Kashii H, Sato A, Hagino Y, Tanaka M, *et al.* (2019): Effects of rapamycin on social interaction deficits and gene expression in mice exposed to valproic acid in utero. *Mol Brain* 12: 3.

191. Christensen J, Grønborg TK, Sørensen MJ, Schendel D, Parner ET, Pedersen LH, Vestergaard M (2013): Prenatal valproate exposure and risk of autism spectrum disorders and childhood autism. *JAMA* 309: 1696.

192. Ageta H, Murayama A, Migishima R, Kida S, Tsuchida K, Yokoyama M, Inokuchi K (2008): Activin in the brain modulates anxiety-related behavior and adult neurogenesis. *PLOS ONE* 3: e1869.

193. Wenderski W, Wang L, Krokhotin A, Walsh JJ, Li H, Shoji H, *et al.* (2020): Loss of the neural-specific BAF subunit ACTL6B relieves repression of early response genes and causes recessive autism. *Proc Natl Acad Sci* 117: 10055–10066.

194. Wu JI, Lessard J, Olave IA, Qiu Z, Ghosh A, Graef IA, Crabtree GR (2007): Regulation of dendritic development by neuron-specific chromatin remodeling complexes. *Neuron* 56: 94–108.

195. Kanetake T, Sassa T, Nojiri K, Sawai M, Hattori S, Miyakawa T, *et al.* (2019): Neural symptoms in a gene knockout mouse model of Sjögren-Larsson syndrome are associated with a decrease in 2-hydroxygalactosylceramide. *FASEB J* 33: 928–941.

196. Naganuma T, Takagi S, Kanetake T, Kitamura T, Hattori S, Miyakawa T, *et al.* (2016): Disruption of the Sjögren-Larsson Syndrome Gene Aldh3a2 in Mice Increases Keratinocyte Growth and Retards Skin Barrier Recovery. *J Biol Chem* 291: 11676–11688.

197. Laurenzi VD, Rogers GR, Hamrock DJ, Marekov LN, Steinert PM, Compton JG, *et al.* (1996): Sjögren–Larsson syndrome is caused by mutations in the fatty aldehyde dehydrogenase gene. *Nat Genet* 12: 52–57.

198. Glynn MW, Elmer BM, Garay PA, Liu X-B, Needleman LA, El-Sabeawy F, McAllister AK (2011): MHCI negatively regulates synapse density during the establishment of cortical connections. *Nat Neurosci* 14: 442–451.

199. Katano T, Fukuda M, Furue H, Yamazaki M, Abe M, Watanabe M, *et al.* (2016): Involvement of brain-enriched guanylate kinase-associated protein (BEGAIN) in chronic pain after peripheral nerve injury. *eNeuro* 3: ENEURO.0110-16.2016.

200. Katano T, Konno K, Takao K, Abe M, Sakimura K, Miyakawa T, *et al.* (2022): Involvement of BEGAIN in memory formation as an excitatory postsynaptic protein in the hippocampus. *NEURO2022 Abstr*.

201. Katano T, Takao K, Abe M, Yamazaki M, Watanabe M, Miyakawa T, *et al.* (2018): Distribution of Caskin1 protein and phenotypic characterization of its knockout mice using a comprehensive behavioral test battery. *Mol Brain* 11: 63.

202. Kaneko-Goto T, Yoshihara S, Miyazaki H, Yoshihara Y (2008): BIG-2 mediates olfactory axon convergence to target glomeruli. *Neuron* 57: 834–846.

203. Fernandez T, Morgan T, Davis N, Klin A, Morris A, Farhi A, *et al.* (2004): Disruption of Contactin 4 (CNTN4) results in developmental delay and other features of 3p deletion syndrome. *Am J Hum Genet* 74: 1286–1293.

204. Yoshioka N, Miyata S, Tamada A, Watanabe Y, Kawasaki A, Kitagawa H, *et al.* (2017): Abnormalities in perineuronal nets and behavior in mice lacking CSGalNAcT1, a key enzyme in chondroitin sulfate synthesis. *Mol Brain* 10: 47.

205. Watanabe Y, Takeuchi K, Higa Onaga S, Sato M, Tsujita M, Abe M, *et al.* (2010): Chondroitin sulfate N-acetylgalactosaminyltransferase-1 is required for normal cartilage development. *Biochem J* 432: 47–55.

206. Ichihara K, Matsumoto A, Nishida H, Kito Y, Shimizu H, Shichino Y, *et al.* (2021): Combinatorial analysis of translation dynamics reveals eIF2 dependence of translation initiation at near-cognate codons. *Nucleic Acids Res* 49: 7298–7317.

207. Itoh M, Yamashita M, Kaneko M, Okuno H, Abe M, Yamazaki M, *et al.* (2018): Deficiency of AMPAR–palmitoylation aggravates seizure susceptibility. *J Neurosci* 38: 10220–10235.

208. Oota-Ishigaki A, Takao K, Yamada D, Sekiguchi M, Itoh M, Koshidata Y, *et al.* (2022): Prolonged contextual fear memory in AMPA receptor palmitoylation-deficient mice. *Neuropsychopharmacology* 47: 2150–2159.

209. Furuse T, Wada Y, Hattori K, Yamada I, Kushida T, Shibukawa Y, *et al.* (2010): Phenotypic characterization of a new Grin1 mutant mouse generated by ENU mutagenesis. *Eur J Neurosci* 31: 1281–1291.

210. Ishikawa K, Yamamoto S, Hattori S, Nishimura N, Tani H, Mito T, *et al.* (2019): Acquired expression of mutant Mitofusin 2 causes progressive neurodegeneration and abnormal behavior. *J Neurosci* 39: 1588–1604.

211. Ishikawa K, Yamamoto S, Hattori S, Nishimura N, Matsumoto H, Miyakawa T, Nakada K (2021): Neuronal degeneration and cognitive impairment can be prevented via the normalization of mitochondrial dynamics. *Pharmacol Res* 163: 105246.

212. Züchner S, Mersiyanova IV, Muglia M, Bissar-Tadmouri N, Rochelle J, Dadali EL, *et al.* (2004): Mutations in the mitochondrial GTPase mitofusin 2 cause Charcot-Marie-Tooth neuropathy type 2A. *Nat Genet* 36: 449–451.

213. Feely SME, Laura M, Siskind CE, Sottile S, Davis M, Gibbons VS, *et al.* (2011): MFN2 mutations cause severe phenotypes in most patients with CMT2A. *Neurology* 76: 1690–1696.

214. Adachi N, Tomonaga S, Tachibana T, Denbow DM, Furuse M (2006): (−)-Epigallocatechin gallate attenuates acute stress responses through GABAergic system in the brain. *Eur J Pharmacol* 531: 171–175.

215. Saito S, Tachibana T, Choi Y-H, Denbow DM, Furuse M (2005): ICV CRF and isolation stress differentially enhance plasma corticosterone concentrations in layer- and meat-type neonatal chicks. *Comp Biochem Physiol A Mol Integr Physiol* 141: 305–309.

216. Yamada M, Yoshida Y, Mori D, Takitoh T, Kengaku M, Umeshima H, *et al.* (2009): Inhibition of calpain increases LIS1 expression and partially rescues in vivo phenotypes in a mouse model of lissencephaly [no. 10]. *Nat Med* 15: 1202–1207.

217. Toba S, Tamura Y, Kumamoto K, Yamada M, Takao K, Hattori S, *et al.* (2013): Post-natal treatment by a blood-brain-barrier permeable calpain inhibitor, SNJ1945 rescued defective function in lissencephaly. *Sci Rep* 3: 1224.

218. Reiner O, Carrozzo R, Shen Y, Wehnert M, Faustinella F, Dobyns WB, *et al.* (1993): Isolation of a Miller–Dicker lissencephaly gene containing G protein β-subunit-like repeats. *Nature* 364: 717–721.

219. Pilz DT, Matsumoto N, Minnerath S, Mills P, Gleeson JG, Allen KM, *et al.* (1998): LIS1 and XLIS (DCX) mutations cause most classical lissencephaly, but different patterns of malformation. *Hum Mol Genet* 7: 2029–2037.

220. Kruse SE, Watt WC, Marcinek DJ, Kapur RP, Schenkman KA, Palmiter RD (2008): Mice with mitochondrial complex I deficiency develop a fatal encephalomyopathy. *Cell Metab* 7: 312–320.

221. van den Heuvel L, Ruitenbeek W, Smeets R, Gelman-Kohan Z, Elpeleg O, Loeffen J, *et al.* (1998): Demonstration of a new pathogenic mutation in human complex I deficiency: A 5-bp duplication in the nuclear gene encoding the 18-kD (AQDQ) subunit. *Am J Hum Genet* 62: 262–268.

222. Petruzzella V, Vergari R, Puzziferri I, Boffoli D, Lamantea E, Zeviani M, Papa S (2001): A nonsense mutation in the NDUFS4 gene encoding the 18 kDa (AQDQ) subunit of complex I abolishes assembly and activity of the complex in a patient with Leigh-like syndrome. *Hum Mol Genet* 10: 529–536.

223. Morishita Y, Yoshioka Y, Takao K, Yamashita K, Yoshikawa T, Itoh N, *et al.* (2011): Postnatal effects of prenatal treatment of amorphous nanosilica. *Toxicol Lett* 205: S285–S286.

224. Moriya Y, Hall SF, Kasahara Y, Hagino Y, Kieffer BL, Uhl GR, *et al.* (2019): Behavioral sensitization and relapse in mu-, delta- and kappa-opioid receptor knockout mice. *6th Congr Asian Coll Neuropsychopharmacol*.

225. Kotajima-Murakami H, Hagihara H, Sato A, Hagino Y, Tanaka M, Katoh Y, *et al.* (2022): Exposure to GABAA receptor antagonist picrotoxin in pregnant mice causes autism-like behaviors and aberrant gene expression in offspring. *Front Psychiatry* 13: 821354.

226. Sato A, Kotajima-Murakami H, Tanaka M, Katoh Y, Ikeda K (2022): Influence of prenatal drug exposure, maternal inflammation, and parental aging on the development of autism spectrum disorder. *Front Psychiatry* 13: 821455.

227. Uyeda A, Onishi K, Hirayama T, Hattori S, Miyakawa T, Yagi T, *et al.* (2020): Suppression of DNA double-strand break formation by DNA polymerase β in active DNA demethylation is required for development of hippocampal pyramidal neurons. *J Neurosci* 40: 9012–9027.

228. McKinnon PJ (2013): Maintaining genome stability in the nervous system. *Nat Neurosci* 16: 1523–1529.

229. Madabhushi R, Pan L, Tsai L-H (2014): DNA Damage and Its Links to Neurodegeneration. *Neuron* 83: 266–282.

230. Tao H, Suzuki M, Kiyonari H, Abe T, Sasaoka T, Ueno N (2009): Mouse prickle1, the homolog of a PCP gene, is essential for epiblast apical-basal polarity. *Proc Natl Acad Sci* 106: 14426–14431.

231. Paemka L, Mahajan VB, Skeie JM, Sowers LP, Ehaideb SN, Gonzalez-Alegre P, *et al.* (2013): PRICKLE1 interaction with SYNAPSIN I reveals a role in autism spectrum disorders. *PLOS ONE* 8: e80737.

232. Bassuk AG, Wallace RH, Buhr A, Buller AR, Afawi Z, Shimojo M, *et al.* (2008): A homozygous mutation in human PRICKLE1 causes an autosomal-recessive progressive myoclonus epilepsy-ataxia syndrome. *Am J Hum Genet* 83: 572–581.

233. Mastrangelo M, Tolve M, Martinelli M, Di Noia SP, Parrini E, Leuzzi V (2018): PRICKLE1-related early onset epileptic encephalopathy. *Am J Med Genet A* 176: 2841–2845.

234. Cukier HN, Dueker ND, Slifer SH, Lee JM, Whitehead PL, Lalanne E, *et al.* (2014): Exome sequencing of extended families with autism reveals genes shared across neurodevelopmental and neuropsychiatric disorders. *Mol Autism* 5: 1.

235. Todd BP, Bassuk AG (2018): A de novo mutation in PRICKLE1 associated with myoclonic epilepsy and autism spectrum disorder. *J Neurogenet* 32: 313–315.

236. Yamamoto, T, Hattori S, Kiyonari H, Nakao K, Miyakawa T, Kubo Y (2014): Comprehensive behavioral test battery analyses of the gene targeted mice of Prrt3, an orphan metabo-tropic receptor. *91st Annu Meet Physiol Soc Jpn Abstr 1P-009*.

237. Ohashi R, Takao K, Miyakawa T, Shiina N (2016): Comprehensive behavioral analysis of RNG105 (Caprin1) heterozygous mice: Reduced social interaction and attenuated response to novelty. *Sci Rep* 6: 20775.

238. Jiang Y, Yuen RKC, Jin X, Wang M, Chen N, Wu X, *et al.* (2013): Detection of clinically relevant genetic variants in autism spectrum disorder by whole-genome sequencing. *Am J Hum Genet* 93: 249–263.

239. Ricobaraza A, Mora-Jimenez L, Puerta E, Sanchez-Carpintero R, Mingorance A, Artieda J, *et al.* (2019): Epilepsy and neuropsychiatric comorbidities in mice carrying a recurrent Dravet syndrome SCN1A missense mutation. *Sci Rep* 9: 14172.

240. Claes L, Del-Favero J, Ceulemans B, Lagae L, Van Broeckhoven C, De Jonghe P (2001): De novo mutations in the sodium-channel gene SCN1A cause severe myoclonic epilepsy of infancy. *Am J Hum Genet* 68: 1327–1332.

241. Marini C, Scheffer IE, Nabbout R, Mei D, Cox K, Dibbens LM, *et al.* (2009): SCN1A duplications and deletions detected in Dravet syndrome: Implications for molecular diagnosis. *Epilepsia* 50: 1670–1678.

242. Shimizu K, Kobayashi Y, Nakatsuji E, Yamazaki M, Shimba S, Sakimura K, Fukada Y (2016): SCOP/PHLPP1β mediates circadian regulation of long-term recognition memory. *Nat Commun* 7: 12926.

243. Oishi K, Okauchi H, Yamamoto S, Higo-Yamamoto S (2020): Dietary natural cocoa ameliorates disrupted circadian rhythms in locomotor activity and sleep-wake cycles in mice with chronic sleep disorders caused by psychophysiological stress. *Nutrition* 75–76: 110751.

244. Sakamoto K, Higo-Yamamoto S, Egi Y, Miyazaki K, Oishi K (2020): Memory dysfunction and anxiety-like behavior in a mouse model of chronic sleep disorders. *Biochem Biophys Res Commun* 529: 175–179.

245. Inoue R, Talukdar G, Takao K, Miyakawa T, Mori H (2018): Dissociated role of D-serine in extinction during consolidation vs. reconsolidation of context conditioned fear. *Front Mol Neurosci* 11: 161.

246. Miya K, Inoue R, Takata Y, Abe M, Natsume R, Sakimura K, *et al.* (2008): Serine racemase is predominantly localized in neurons in mouse brain. *J Comp Neurol* 510: 641–654.

247. Goltsov AY, Loseva JG, Andreeva TV, Grigorenko AP, Abramova LI, Kaleda VG, *et al.* (2006): Polymorphism in the 5′-promoter region of serine racemase gene in schizophrenia. *Mol Psychiatry* 11: 325–326.

248. Labrie V, Fukumura R, Rastogi A, Fick LJ, Wang W, Boutros PC, *et al.* (2009): Serine racemase is associated with schizophrenia susceptibility in humans and in a mouse model. *Hum Mol Genet* 18: 3227–3243.

249. Watanabe Y, Katayama N, Takeuchi K, Togano T, Itoh R, Sato M, *et al.* (2013): Point mutation in syntaxin-1A causes abnormal vesicle recycling, behaviors, and short term plasticity. *J Biol Chem* 288: 34906–34919.

250. Yamada I, Kushida T, Miura I, Furuse T, Tamura M (2021): Analysis of the correlation between social behavior and biochemical data using a telemetry system. *Exp Anim* 70: S75.

251. O’Roak BJ, Deriziotis P, Lee C, Vives L, Schwartz JJ, Girirajan S, *et al.* (2011): Exome sequencing in sporadic autism spectrum disorders identifies severe de novo mutations. *Nat Genet* 43: 585–589.

252. Andoh C, Nishitani N, Hashimoto E, Nagai Y, Takao K, Miyakawa T, *et al.* (2019): TRPM2 confers susceptibility to social stress but is essential for behavioral flexibility. *Brain Res* 1704: 68–77.

253. Xu C, Li PP, Cooke RG, Parikh SV, Wang K, Kennedy JL, Warsh JJ (2009): TRPM2 variants and bipolar disorder risk: confirmation in a family-based association study. *Bipolar Disord* 11: 1–10.

254. Xu C, Macciardi F, Li PP, Yoon I-S, Cooke RG, Hughes B, *et al.* (2006): Association of the putative susceptibility gene, transient receptor potential protein melastatin type 2, with bipolar disorder. *Am J Med Genet B Neuropsychiatr Genet* 141B: 36–43.

255. Shimohata A, Ishihara K, Hattori S, Miyamoto H, Morishita H, Ornthanalai G, *et al.* (2017): Ts1Cje Down syndrome model mice exhibit environmental stimuli-triggered locomotor hyperactivity and sociability concurrent with increased flux through central dopamine and serotonin metabolism. *Exp Neurol* 293: 1–12.

256. Sago H, Carlson EJ, Smith DJ, Kilbridge J, Rubin EM, Mobley WC, *et al.* (1998): Ts1Cje, a partial trisomy 16 mouse model for Down syndrome, exhibits learning and behavioral abnormalities. *Proc Natl Acad Sci* 95: 6256–6261.

257. Auerbach BD, Osterweil EK, Bear MF (2011): Mutations causing syndromic autism define an axis of synaptic pathophysiology. *Nature* 480: 63–68.

258. Ehninger D, Han S, Shilyansky C, Zhou Y, Li W, Kwiatkowski DJ, *et al.* (2008): Reversal of learning deficits in a Tsc2+/− mouse model of tuberous sclerosis. *Nat Med* 14: 843–848.

259. Sugiura H, Shimada T, Moriya-Ito K, Goto J-I, Fujiwara H, Ishii R, *et al.* (2022): A farnesyltransferase inhibitor restores cognitive deficits in Tsc2+/- mice through inhibition of Rheb1. *J Neurosci* 42: 2598–2612.

260. Miura K, Kishino T, Li E, Webber H, Dikkes P, Holmes GL, Wagstaff J (2002): Neurobehavioral and electroencephalographic abnormalities in Ube3a maternal-deficient mice. *Neurobiol Dis* 9: 149–159.

261. Williams CA, Zori RT, Hendrickson J, Stalker H, Marum T, Whidden E, Driscoll DJ (1995): Angelman syndrome. *Curr Probl Pediatr* 25: 216–231.

262. Sato DX, Inoue YU, Morimoto Y, Inoue T, Kuga N, Sasaki T, *et al.* (2021, August 17): Humanized substitutions of Vmat1 in mice alter amygdala-dependent behaviors associated with the evolution of anxiety. bioRxiv, p 2021.05.18.444749.

263. Lohoff FW, Dahl JP, Ferraro TN, Arnold SE, Gallinat J, Sander T, Berrettini WH (2006): Variations in the vesicular monoamine transporter 1 gene (VMAT1/SLC18A1) are associated with bipolar I disorder. *Neuropsychopharmacology* 31: 2739–2747.

264. Lohoff FW, Lautenschlager M, Mohr J, Ferraro TN, Sander T, Gallinat J (2008): Association between variation in the vesicular monoamine transporter 1 gene on chromosome 8p and anxiety-related personality traits. *Neurosci Lett* 434: 41–45.

265. Vaht M, Kiive E, Veidebaum T, Harro J (2016): A functional vesicular monoamine transporter 1 (VMAT1) gene variant is associated with affect and the prevalence of anxiety, affective, and alcohol use disorders in a longitudinal population-representative birth cohort study. *Int J Neuropsychopharmacol* 19: pyw013.

266. Takagi T, Nishizaki Y, Matsui F, Wakamatsu N, Higashi Y (2015): De novo inbred heterozygous Zeb2/Sip1 mutant mice uniquely generated by germ-line conditional knockout exhibit craniofacial, callosal and behavioral defects associated with Mowat–Wilson syndrome. *Hum Mol Genet* 24: 6390–6402.

267. Cacheux V, Dastot-Le Moal F, Kääriäinen H, Bondurand N, Rintala R, Boissier B, *et al.* (2001): Loss-of-function mutations in SIP1 Smad interacting protein 1 result in a syndromic Hirschsprung disease. *Hum Mol Genet* 10: 1503–1510.

268. Wakamatsu N, Yamada Y, Yamada K, Ono T, Nomura N, Taniguchi H, *et al.* (2001): Mutations in SIP1, encoding Smad interacting protein-1, cause a form of Hirschsprung disease. *Nat Genet* 27: 369–370.

269. Komine Y, Takao K, Miyakawa T, Yamamori T (2012): Behavioral Abnormalities Observed in Zfhx2-Deficient Mice. *PLOS ONE* 7: e53114.
